# 2-(1*H*-Benzotriazol-1-yl)-1-(3-methoxy­benzo­yl)ethyl isonicotinate

**DOI:** 10.1107/S1600536809001196

**Published:** 2009-01-23

**Authors:** Wei Wang, Zhen-Hua Mei

**Affiliations:** a College of Life Science and Pharmaceutical Engineering, Nanjing University of Technology, 210009 Nanjing, Jiangsu, People’s Republic of China

## Abstract

In the title compound, C_22_H_18_N_4_O_4_, mol­ecules are linked to each other by C—H⋯N and C—H⋯O inter­molecular hydrogen-bonding inter­actions. The crystal packing is further stabilized by C—H⋯π, and π–π inter­actions with a distance of 3.783 (3) Å between the centroids of the benzene rings of the benzotriazole system.

## Related literature

For general background on benzotriazole and its derivatives, see: Chen & Wu (2005 ▶). For details of the synthesis, see: Wan *et al.* (2006 ▶). For bond-length data, see: Allen *et al.* (1987 ▶).
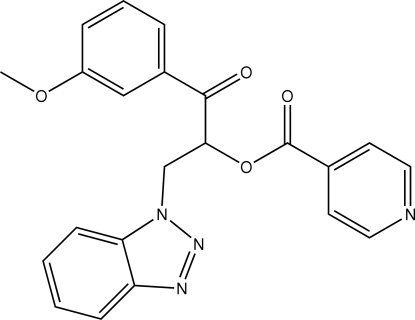


## Experimental

### 

#### Crystal data

C_22_H_18_N_4_O_4_
*M* *_r_* = 402.40
Triclinic, 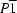

*a* = 9.4839 (18) Å
*b* = 10.3611 (19) Å
*c* = 11.276 (2) Å
α = 109.342 (3)°
β = 102.664 (3)°
γ = 97.985 (3)°
*V* = 992.8 (3) Å^3^
*Z* = 2
Mo *K*α radiation
μ = 0.10 mm^−1^
*T* = 293 (2) K
0.33 × 0.16 × 0.08 mm

#### Data collection

Siemens SMART 1000 CCD area-detector diffractometer
Absorption correction: multi-scan (*SADABS*; Sheldrick, 1996 ▶) *T*_min_ = 0.969, *T*_max_ = 0.992
5605 measured reflections
3819 independent reflections
2822 reflections with *I* > 2σ(*I*)
*R*_int_ = 0.013

#### Refinement

*R*[*F* ^2^ > 2σ(*F* ^2^)] = 0.046
*wR*(*F* ^2^) = 0.119
*S* = 1.04
3819 reflections
271 parameters
H-atom parameters constrained
Δρ_max_ = 0.15 e Å^−3^
Δρ_min_ = −0.19 e Å^−3^

### 

Data collection: *SMART* (Siemens, 1996 ▶); cell refinement: *SAINT* (Siemens, 1996 ▶); data reduction: *SAINT*; program(s) used to solve structure: *SHELXS97* (Sheldrick, 2008 ▶); program(s) used to refine structure: *SHELXL97* (Sheldrick, 2008 ▶); molecular graphics: *SHELXTL* (Sheldrick, 2008 ▶); software used to prepare material for publication: *SHELXTL*, *PARST* (Nardelli, 1995 ▶) and *PLATON* (Spek, 2003 ▶).

## Supplementary Material

Crystal structure: contains datablocks global, I. DOI: 10.1107/S1600536809001196/at2705sup1.cif

Structure factors: contains datablocks I. DOI: 10.1107/S1600536809001196/at2705Isup2.hkl

Additional supplementary materials:  crystallographic information; 3D view; checkCIF report

## Figures and Tables

**Table 1:** Hydrogen-bond geometry (Å, °) *Cg*3 and *Cg*4 are the centroids of the C1–C6 and C10–C15 benzene rings, respectively.

*D*—H⋯*A*	*D*—H	H⋯*A*	*D*⋯*A*	*D*—H⋯*A*
C3—H3*B*⋯N3^i^	0.93	2.48	3.328 (3)	151
C9—H9*B*⋯O1^ii^	0.97	2.56	3.471 (2)	157
C15—H15*A*⋯O1^ii^	0.93	2.46	3.368 (3)	164
C20—H20*A*⋯*Cg*3^iii^	0.93	2.85	3.767	168
C22—H22*C*⋯*Cg*4^iv^	0.96	2.87	3.530	127

Symmetry codes: (i) 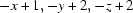; (ii) 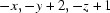; (iii) 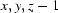; (iv) 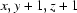.

**Fig. 1.**
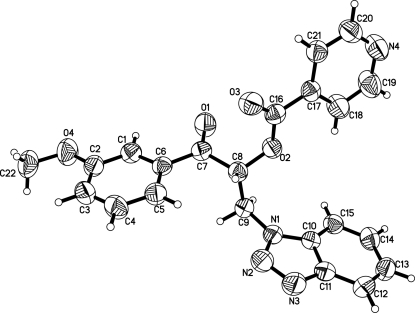
The structure of the compound (I) showing 50% probability displacement ellipsoids and the atom numbering scheme.

**Fig. 2.**
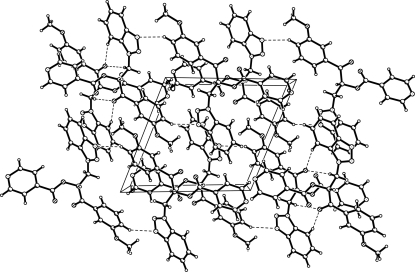
A packing diagram of (I), viewed down the a axis. Hydrogen bonds are indicated by dashed lines.

## supplementary crystallographic information

### Comment

Benzotriazole and its derivatives exhibit an excellent pharmacological activities, such as antifungal, antitumor and antineoplastic activities (Chen & Wu, 2005). In order to find a new benzotriazole compound with higher bioactivity, the title compound, (I) (Fig. 1), was synthesized and its crystal structure was presented here.

In (I), all bond lengths and angles are within normal ranges (Allen *et al.*, 1987). The benzotriazole ring is mostly planar with a dihedral angle of 1.75 (1)° between the N1—N3/C10/C11 triazole and C10—C15 benzene rings. The mean plane makes dihedral angles of 70.93 (1) and 19.8 (1)° with the N4/C17—C21 pyridine and C1—C6 benzene rings, respectively, and the dihedral angle between the latter two aromatic rings is 80.08 (1)°.

In the crystal structure, molecules of (I) are linked to each other by C3—H3B···N3, and further linked by C—H···O intermolecular hydrogen bonding interactions (C9—H9B···O1 and C15—H15A···O1), and stabilized by C—H···π interactions (Table 1). The distances of 3.783 Å between the centroids of the rings C10—C15 related by the symmetry code (-*x*, 1 - *y*, 1 - *z*) suggests a possible π–π interaction.

### Experimental

The title compound was prepared according to the literature method of Wan *et al.* (20067). Single crystals suitable for X-ray diffraction were obtained by slow evaporation of an ethyl acetate solution at room temperature over a period of one week.

### Refinement

All H atoms were located in difference Fourier maps and constrained to ride on their parent atoms, with C—H distances in the range 0.93–0.97 Å, and with *U*_iso_(H) = 1.2 *U*_eq_(C) and 1.5 *U*_eq_(methyl C).

### Crystal data

**Table:**

C_22_H_18_N_4_O_4_	*Z* = 2
*M**_r_* = 402.40	*F*(000) = 420
Triclinic, *P*1	*D*_x_ = 1.346 Mg m^−^^3^
Hall symbol: -P 1	Mo *K*α radiation, λ = 0.71073 Å
*a* = 9.4839 (18) Å	Cell parameters from 1711 reflections
*b* = 10.3611 (19) Å	θ = 2.6–24.7°
*c* = 11.276 (2) Å	µ = 0.10 mm^−^^1^
α = 109.342 (3)°	*T* = 293 K
β = 102.664 (3)°	Plate, colourless
γ = 97.985 (3)°	0.33 × 0.16 × 0.08 mm
*V* = 992.8 (3) Å^3^	

### Data collection

**Table:**

Siemens SMART 1000 CCD area-detector diffractometer	3819 independent reflections
Radiation source: fine-focus sealed tube	2822 reflections with *I* > 2σ(*I*)
graphite	*R*_int_ = 0.013
Detector resolution: 8.33 pixels mm^-1^	θ_max_ = 26.1°, θ_min_ = 2.0°
ω scans	*h* = −11→11
Absorption correction: multi-scan (*SADABS*; Sheldrick, 1996)	*k* = −12→10
*T*_min_ = 0.969, *T*_max_ = 0.992	*l* = −12→13
5605 measured reflections	

### Refinement

**Table:**

Refinement on *F*^2^	Primary atom site location: structure-invariant direct methods
Least-squares matrix: full	Secondary atom site location: difference Fourier map
*R*[*F*^2^ > 2σ(*F*^2^)] = 0.046	Hydrogen site location: inferred from neighbouring sites
*wR*(*F*^2^) = 0.119	H-atom parameters constrained
*S* = 1.04	*w* = 1/[σ^2^(*F*_o_^2^) + (0.0502*P*)^2^ + 0.1574*P*] where *P* = (*F*_o_^2^ + 2*F*_c_^2^)/3
3819 reflections	(Δ/σ)_max_ < 0.001
271 parameters	Δρ_max_ = 0.15 e Å^−^^3^
0 restraints	Δρ_min_ = −0.19 e Å^−^^3^

### Special details

**Table:**

Geometry. All e.s.d.'s (except the e.s.d. in the dihedral angle between two l.s. planes) are estimated using the full covariance matrix. The cell e.s.d.'s are taken into account individually in the estimation of e.s.d.'s in distances, angles and torsion angles; correlations between e.s.d.'s in cell parameters are only used when they are defined by crystal symmetry. An approximate (isotropic) treatment of cell e.s.d.'s is used for estimating e.s.d.'s involving l.s. planes.
Refinement. Refinement of *F*^2^ against ALL reflections. The weighted *R*-factor *wR* and goodness of fit *S* are based on *F*^2^, conventional *R*-factors *R* are based on *F*, with *F* set to zero for negative *F*^2^. The threshold expression of *F*^2^ > σ(*F*^2^) is used only for calculating *R*-factors(gt) *etc*. and is not relevant to the choice of reflections for refinement. *R*-factors based on *F*^2^ are statistically about twice as large as those based on *F*, and *R*- factors based on ALL data will be even larger.

### Fractional atomic coordinates and isotropic or equivalent isotropic displacement parameters (Å^2^)

**Table:**

	*x*	*y*	*z*	*U*_iso_*/*U*_eq_	
O2	0.21549 (13)	0.94145 (12)	0.45684 (11)	0.0511 (3)	
O3	0.40290 (15)	1.11047 (14)	0.47141 (13)	0.0639 (4)	
N1	0.22089 (16)	0.73861 (15)	0.58935 (14)	0.0498 (4)	
C7	0.23974 (18)	1.12532 (18)	0.66726 (18)	0.0474 (4)	
C6	0.31816 (18)	1.19911 (18)	0.80934 (17)	0.0457 (4)	
C17	0.23003 (19)	0.97219 (18)	0.26265 (17)	0.0481 (4)	
C1	0.2728 (2)	1.31472 (19)	0.88006 (18)	0.0526 (5)	
H1A	0.1952	1.3441	0.8379	0.063*	
C16	0.29418 (19)	1.01773 (19)	0.40669 (18)	0.0487 (4)	
O1	0.15281 (15)	1.17332 (14)	0.60783 (13)	0.0691 (4)	
C8	0.26559 (19)	0.98062 (18)	0.59616 (17)	0.0474 (4)	
H8A	0.3712	0.9811	0.6233	0.057*	
N2	0.34386 (18)	0.72584 (18)	0.66902 (16)	0.0642 (5)	
C10	0.16172 (19)	0.61663 (18)	0.48299 (17)	0.0457 (4)	
C21	0.2851 (2)	1.0500 (2)	0.19695 (19)	0.0556 (5)	
H21A	0.3604	1.1310	0.2422	0.067*	
C9	0.1742 (2)	0.86971 (18)	0.62674 (18)	0.0498 (4)	
H9A	0.1837	0.9040	0.7199	0.060*	
H9B	0.0704	0.8535	0.5805	0.060*	
C15	0.0363 (2)	0.57215 (19)	0.37579 (18)	0.0524 (5)	
H15A	−0.0264	0.6310	0.3634	0.063*	
N3	0.36612 (19)	0.60040 (18)	0.61744 (17)	0.0687 (5)	
O4	0.28449 (18)	1.49647 (15)	1.07256 (15)	0.0812 (5)	
C11	0.2550 (2)	0.52895 (19)	0.50226 (18)	0.0522 (5)	
C5	0.4361 (2)	1.1570 (2)	0.87303 (19)	0.0565 (5)	
H5A	0.4675	1.0791	0.8273	0.068*	
C2	0.3411 (2)	1.38696 (19)	1.01200 (19)	0.0551 (5)	
N4	0.1207 (2)	0.8890 (2)	−0.00867 (17)	0.0771 (5)	
C3	0.4587 (2)	1.3456 (2)	1.0743 (2)	0.0626 (5)	
H3B	0.5059	1.3944	1.1632	0.075*	
C12	0.2266 (2)	0.3910 (2)	0.4131 (2)	0.0615 (5)	
H12A	0.2887	0.3316	0.4253	0.074*	
C14	0.0107 (2)	0.4364 (2)	0.28959 (19)	0.0604 (5)	
H14A	−0.0717	0.4024	0.2164	0.072*	
C4	0.5054 (2)	1.2319 (2)	1.0041 (2)	0.0670 (6)	
H4B	0.5854	1.2052	1.0460	0.080*	
C13	0.1044 (2)	0.3469 (2)	0.3078 (2)	0.0627 (5)	
H13A	0.0826	0.2555	0.2466	0.075*	
C20	0.2260 (2)	1.0049 (2)	0.0634 (2)	0.0680 (6)	
H20A	0.2622	1.0593	0.0207	0.082*	
C19	0.0693 (3)	0.8157 (3)	0.0559 (2)	0.0825 (7)	
H19A	−0.0042	0.7339	0.0076	0.099*	
C22	0.3532 (3)	1.5758 (2)	1.2089 (2)	0.0840 (7)	
H22A	0.3031	1.6493	1.2391	0.126*	
H22B	0.4555	1.6163	1.2223	0.126*	
H22C	0.3471	1.5151	1.2570	0.126*	
C18	0.1180 (2)	0.8531 (2)	0.1897 (2)	0.0695 (6)	
H18A	0.0762	0.7991	0.2302	0.083*	

### Atomic displacement parameters (Å^2^)

**Table:**

	*U*^11^	*U*^22^	*U*^33^	*U*^12^	*U*^13^	*U*^23^
O2	0.0529 (7)	0.0520 (7)	0.0458 (7)	0.0072 (6)	0.0145 (6)	0.0166 (6)
O3	0.0558 (8)	0.0645 (9)	0.0632 (9)	−0.0020 (7)	0.0069 (7)	0.0265 (7)
N1	0.0547 (9)	0.0462 (9)	0.0449 (9)	0.0113 (7)	0.0066 (7)	0.0176 (7)
C7	0.0417 (9)	0.0488 (10)	0.0528 (11)	0.0117 (8)	0.0111 (8)	0.0213 (8)
C6	0.0435 (9)	0.0445 (10)	0.0490 (11)	0.0063 (8)	0.0119 (8)	0.0196 (8)
C17	0.0463 (10)	0.0511 (11)	0.0494 (11)	0.0166 (8)	0.0176 (8)	0.0172 (8)
C1	0.0477 (10)	0.0533 (11)	0.0565 (12)	0.0135 (8)	0.0109 (9)	0.0219 (9)
C16	0.0473 (10)	0.0476 (10)	0.0546 (11)	0.0149 (9)	0.0175 (9)	0.0199 (9)
O1	0.0717 (9)	0.0700 (9)	0.0587 (9)	0.0335 (8)	0.0025 (7)	0.0186 (7)
C8	0.0464 (10)	0.0486 (10)	0.0451 (10)	0.0114 (8)	0.0100 (8)	0.0165 (8)
N2	0.0651 (10)	0.0611 (11)	0.0550 (10)	0.0141 (8)	−0.0021 (8)	0.0197 (8)
C10	0.0511 (10)	0.0447 (10)	0.0426 (10)	0.0100 (8)	0.0128 (8)	0.0187 (8)
C21	0.0563 (11)	0.0538 (11)	0.0557 (12)	0.0126 (9)	0.0174 (9)	0.0185 (9)
C9	0.0546 (10)	0.0456 (10)	0.0479 (10)	0.0106 (8)	0.0146 (8)	0.0162 (8)
C15	0.0529 (11)	0.0565 (11)	0.0493 (11)	0.0154 (9)	0.0121 (9)	0.0221 (9)
N3	0.0723 (11)	0.0633 (11)	0.0633 (11)	0.0238 (9)	0.0017 (9)	0.0226 (9)
O4	0.0977 (11)	0.0704 (10)	0.0664 (10)	0.0311 (9)	0.0243 (9)	0.0088 (8)
C11	0.0577 (11)	0.0528 (11)	0.0476 (11)	0.0157 (9)	0.0101 (9)	0.0226 (9)
C5	0.0577 (11)	0.0552 (11)	0.0558 (12)	0.0196 (9)	0.0119 (9)	0.0196 (9)
C2	0.0593 (11)	0.0491 (11)	0.0535 (12)	0.0076 (9)	0.0178 (9)	0.0154 (9)
N4	0.0788 (12)	0.0912 (14)	0.0529 (11)	0.0082 (11)	0.0183 (10)	0.0213 (10)
C3	0.0667 (13)	0.0630 (13)	0.0483 (12)	0.0062 (10)	0.0070 (10)	0.0178 (10)
C12	0.0742 (13)	0.0531 (12)	0.0641 (13)	0.0248 (10)	0.0225 (11)	0.0245 (10)
C14	0.0603 (12)	0.0626 (13)	0.0485 (11)	0.0075 (10)	0.0118 (9)	0.0135 (9)
C4	0.0646 (13)	0.0745 (14)	0.0564 (13)	0.0205 (11)	0.0018 (10)	0.0258 (11)
C13	0.0761 (14)	0.0468 (11)	0.0595 (13)	0.0099 (10)	0.0231 (11)	0.0120 (9)
C20	0.0751 (14)	0.0726 (15)	0.0586 (13)	0.0117 (12)	0.0235 (11)	0.0266 (11)
C19	0.0799 (16)	0.0866 (17)	0.0563 (14)	−0.0113 (13)	0.0136 (12)	0.0116 (12)
C22	0.1063 (19)	0.0643 (14)	0.0660 (15)	0.0046 (13)	0.0360 (14)	0.0037 (11)
C18	0.0672 (13)	0.0733 (14)	0.0575 (13)	−0.0036 (11)	0.0170 (11)	0.0194 (11)

### Geometric parameters (Å, °)

**Table:**

O2—C16	1.349 (2)	C15—H15A	0.9300
O2—C8	1.434 (2)	N3—C11	1.375 (2)
O3—C16	1.204 (2)	O4—C2	1.363 (2)
N1—N2	1.359 (2)	O4—C22	1.428 (2)
N1—C10	1.362 (2)	C11—C12	1.396 (3)
N1—C9	1.445 (2)	C5—C4	1.375 (3)
C7—O1	1.212 (2)	C5—H5A	0.9300
C7—C6	1.488 (2)	C2—C3	1.382 (3)
C7—C8	1.531 (2)	N4—C19	1.327 (3)
C6—C1	1.384 (2)	N4—C20	1.329 (3)
C6—C5	1.395 (2)	C3—C4	1.376 (3)
C17—C18	1.382 (3)	C3—H3B	0.9300
C17—C21	1.385 (3)	C12—C13	1.361 (3)
C17—C16	1.487 (2)	C12—H12A	0.9300
C1—C2	1.378 (3)	C14—C13	1.403 (3)
C1—H1A	0.9300	C14—H14A	0.9300
C8—C9	1.519 (2)	C4—H4B	0.9300
C8—H8A	0.9800	C13—H13A	0.9300
N2—N3	1.304 (2)	C20—H20A	0.9300
C10—C11	1.387 (2)	C19—C18	1.378 (3)
C10—C15	1.392 (2)	C19—H19A	0.9300
C21—C20	1.375 (3)	C22—H22A	0.9600
C21—H21A	0.9300	C22—H22B	0.9600
C9—H9A	0.9700	C22—H22C	0.9600
C9—H9B	0.9700	C18—H18A	0.9300
C15—C14	1.371 (3)		
			
C16—O2—C8	115.78 (13)	C2—O4—C22	117.93 (18)
N2—N1—C10	109.89 (14)	N3—C11—C10	108.94 (16)
N2—N1—C9	118.99 (14)	N3—C11—C12	130.21 (18)
C10—N1—C9	131.12 (15)	C10—C11—C12	120.83 (17)
O1—C7—C6	122.39 (16)	C4—C5—C6	119.33 (18)
O1—C7—C8	119.34 (16)	C4—C5—H5A	120.3
C6—C7—C8	118.21 (15)	C6—C5—H5A	120.3
C1—C6—C5	119.12 (17)	O4—C2—C1	115.94 (18)
C1—C6—C7	118.39 (15)	O4—C2—C3	124.37 (18)
C5—C6—C7	122.49 (16)	C1—C2—C3	119.69 (18)
C18—C17—C21	117.82 (18)	C19—N4—C20	116.16 (19)
C18—C17—C16	122.72 (18)	C4—C3—C2	119.54 (19)
C21—C17—C16	119.45 (17)	C4—C3—H3B	120.2
C2—C1—C6	120.96 (17)	C2—C3—H3B	120.2
C2—C1—H1A	119.5	C13—C12—C11	117.29 (18)
C6—C1—H1A	119.5	C13—C12—H12A	121.4
O3—C16—O2	123.70 (17)	C11—C12—H12A	121.4
O3—C16—C17	124.81 (18)	C15—C14—C13	122.40 (19)
O2—C16—C17	111.49 (15)	C15—C14—H14A	118.8
O2—C8—C9	106.06 (13)	C13—C14—H14A	118.8
O2—C8—C7	110.80 (14)	C5—C4—C3	121.34 (19)
C9—C8—C7	109.81 (14)	C5—C4—H4B	119.3
O2—C8—H8A	110.0	C3—C4—H4B	119.3
C9—C8—H8A	110.0	C12—C13—C14	121.34 (18)
C7—C8—H8A	110.0	C12—C13—H13A	119.3
N3—N2—N1	109.04 (15)	C14—C13—H13A	119.3
N1—C10—C11	104.25 (15)	N4—C20—C21	124.4 (2)
N1—C10—C15	133.49 (16)	N4—C20—H20A	117.8
C11—C10—C15	122.22 (16)	C21—C20—H20A	117.8
C20—C21—C17	118.61 (19)	N4—C19—C18	124.2 (2)
C20—C21—H21A	120.7	N4—C19—H19A	117.9
C17—C21—H21A	120.7	C18—C19—H19A	117.9
N1—C9—C8	111.93 (14)	O4—C22—H22A	109.5
N1—C9—H9A	109.2	O4—C22—H22B	109.5
C8—C9—H9A	109.2	H22A—C22—H22B	109.5
N1—C9—H9B	109.2	O4—C22—H22C	109.5
C8—C9—H9B	109.2	H22A—C22—H22C	109.5
H9A—C9—H9B	107.9	H22B—C22—H22C	109.5
C14—C15—C10	115.92 (17)	C19—C18—C17	118.8 (2)
C14—C15—H15A	122.0	C19—C18—H18A	120.6
C10—C15—H15A	122.0	C17—C18—H18A	120.6
N2—N3—C11	107.87 (15)		
			
O1—C7—C6—C1	10.4 (3)	N1—C10—C15—C14	−177.61 (19)
C8—C7—C6—C1	−166.80 (16)	C11—C10—C15—C14	−0.4 (3)
O1—C7—C6—C5	−168.84 (18)	N1—N2—N3—C11	−0.5 (2)
C8—C7—C6—C5	13.9 (3)	N2—N3—C11—C10	0.6 (2)
C5—C6—C1—C2	−0.9 (3)	N2—N3—C11—C12	−177.9 (2)
C7—C6—C1—C2	179.82 (17)	N1—C10—C11—N3	−0.4 (2)
C8—O2—C16—O3	2.3 (2)	C15—C10—C11—N3	−178.31 (17)
C8—O2—C16—C17	−177.93 (13)	N1—C10—C11—C12	178.28 (18)
C18—C17—C16—O3	170.73 (19)	C15—C10—C11—C12	0.4 (3)
C21—C17—C16—O3	−8.3 (3)	C1—C6—C5—C4	−0.6 (3)
C18—C17—C16—O2	−9.0 (2)	C7—C6—C5—C4	178.69 (18)
C21—C17—C16—O2	172.00 (15)	C22—O4—C2—C1	−179.05 (18)
C16—O2—C8—C9	−172.79 (13)	C22—O4—C2—C3	1.4 (3)
C16—O2—C8—C7	68.10 (18)	C6—C1—C2—O4	−178.11 (17)
O1—C7—C8—O2	18.0 (2)	C6—C1—C2—C3	1.5 (3)
C6—C7—C8—O2	−164.64 (14)	O4—C2—C3—C4	178.9 (2)
O1—C7—C8—C9	−98.8 (2)	C1—C2—C3—C4	−0.6 (3)
C6—C7—C8—C9	78.53 (19)	N3—C11—C12—C13	178.3 (2)
C10—N1—N2—N3	0.3 (2)	C10—C11—C12—C13	−0.1 (3)
C9—N1—N2—N3	−179.98 (16)	C10—C15—C14—C13	0.2 (3)
N2—N1—C10—C11	0.1 (2)	C6—C5—C4—C3	1.5 (3)
C9—N1—C10—C11	−179.61 (17)	C2—C3—C4—C5	−0.9 (3)
N2—N1—C10—C15	177.64 (19)	C11—C12—C13—C14	−0.1 (3)
C9—N1—C10—C15	−2.1 (3)	C15—C14—C13—C12	0.0 (3)
C18—C17—C21—C20	−0.1 (3)	C19—N4—C20—C21	1.4 (3)
C16—C17—C21—C20	178.95 (17)	C17—C21—C20—N4	−1.5 (3)
N2—N1—C9—C8	79.2 (2)	C20—N4—C19—C18	0.2 (4)
C10—N1—C9—C8	−101.1 (2)	N4—C19—C18—C17	−1.7 (4)
O2—C8—C9—N1	74.36 (17)	C21—C17—C18—C19	1.6 (3)
C7—C8—C9—N1	−165.87 (14)	C16—C17—C18—C19	−177.42 (19)

### Hydrogen-bond geometry (Å, °)

**Table:**

*D*—H···*A*	*D*—H	H···*A*	*D*···*A*	*D*—H···*A*
C3—H3B···N3^i^	0.93	2.48	3.328 (3)	151
C9—H9B···O1^ii^	0.97	2.56	3.471 (2)	157
C15—H15A···O1^ii^	0.93	2.46	3.368 (3)	164
C20—H20A···Cg3^iii^	0.93	2.85	3.767	168
C22—H22C···Cg4^iv^	0.96	2.87	3.530	127

Symmetry codes: (i) −*x*+1, −*y*+2, −*z*+2; (ii) −*x*, −*y*+2, −*z*+1; (iii) *x*, *y*, *z*−1; (iv) *x*, *y*+1, *z*+1.
